# Relationship between device acceptance and patient-reported outcomes in Left Ventricular Assist Device (LVAD) recipients

**DOI:** 10.1038/s41598-019-47324-z

**Published:** 2019-07-25

**Authors:** Crispino Tosto, Luigi Adamo, Heidi Craddock, Maria Di Blasi, Rosario Girgenti, Francesco Clemenza, Robert M. Carney, Gregory Ewald

**Affiliations:** 1 From the University of Palermo, Department of Psychological and Educational Sciences, Palermo, Italy; 2 From the Cardiovascular Division, Department of Medicine, Washington University School of Medicine, St. Louis, MO USA; 3 From the Heart Failure Unit, ISMETT, Palermo, Italy; 4 From the Department of Psychiatry, Washington University School of Medicine, St Louis, MO USA

**Keywords:** Cardiac device therapy, Outcomes research, Heart failure, Quality of life

## Abstract

The number of Left Ventricular Assist Devices (LVADs) implanted each year is rising. Nevertheless, there are minimal data on device acceptance after LVAD implant, and on its relationship with patient-reported outcomes. We designed a cross-sectional study to address this knowledge gap and test the hypothesis that low device acceptance is associated with poorer quality of life, depression and anxiety. Self-report questionnaires were administered to assess quality of life (12-item Kansas City Cardiomyopathy Questionnaire quality of life subscale), level of anxiety (7-item Generalized Anxiety Disorder; GAD-7), level of depression (9-item Patient Health Questionnaire; PHQ-9) and device acceptance (Florida Patient Acceptance Survey; FPAS) to 101 consecutive patients presenting to LVAD clinic. Regression analysis showed a strong correlation between device acceptance and both psychological distress (p < 0.001) and quality of life (p < 0.001). Analysis of the sub-scales of the FPAS showed that patients had significant body image concerns, but return to function and device-related distress were the main drivers of the observed correlation between device acceptance and patient well-being. Younger age was associated with lower device acceptance (r = 0.36, p < 0.001) and lower quality of life (r = 0.54, p < 0.001). These findings suggest that interventions targeting device acceptance should be explored to improve outcomes in LVAD recipients.

## Introduction

The few small studies that have investigated the impact of LVAD therapy on psychological distress^1^ have generally found that LVAD recipients experience symptoms of depression and anxiety immediately after implant^2^, but their psychological distress diminishes over time^3–6^. However, one study reported that moderate symptoms of anxiety and depression may remain at least six months after the implant in spite of improved indices of quality of life (QoL)^7^. Clearly, this question needs further study.

There is also very little data on patients’ acceptance of the implanted device, and all of the information available comes from very small studies (<30 patients). Most have found that patients appear to be satisfied with the LVAD and tend to accept it as an essential component of their own bodies and lives^8–10^. However, one study found that LVAD recipients positively evaluated their experience with the device immediately after surgery but tended to be less satisfied with the device as time from the implant increased due to worsening in physical functioning^11^.

There is also minimal information about the impact that the LVAD, which is partially visible, has on patients’ perception of their body image^10,12^. Chapman *et al*. studied 6 patients who reported that LVAD implant was an upsetting event, due to sudden and significant body changes caused by the device^12^. In a sample of 9 patients, Marcuccilli *et al*. found that even when patients came to terms with the modifications that the presence of the LVAD had on their own body, they continued to worry about appearing “normal” in public^10^.

In summary, despite the rapidly growing number of LVAD implants, there are limited and contradictory data about patients’ device acceptance, and no data about the relationship between patients’ device acceptance and the psychological well-being and QoL of LVAD recipients. Moreover, more needs to be known about the impact that partially externalized LVADs have on body-image perception.

We hypothesized that worse levels of device acceptance, especially with regard to body-image, would be associated with poorer QoL and higher levels of depression/anxiety, and could therefore be identified as potential therapeutic targets to improve outcomes post LVAD. To test this hypothesis, we designed a cross-sectional observational cohort study of consecutive patients presenting to the outpatient LVAD clinic of Barnes Jewish Hospital.

## Materials and Method

### Study population and design

Between July 2016 and December 2016, questionnaires were administered to patients presenting for outpatient follow-up to assess QoL, levels of anxiety and depression, and device acceptance. All patients who were older than 18, had no cognitive impairment and were clinically stable were administered the questionnaires. The instruments were not administered at subsequent visits. Data were collected on a cohort of 101 consecutive patients meeting study criteria. All patients provided informed consent, the study was reviewed and approved by the Washington University Institutional Review Board and all data was collected in accordance with relevant guidelines and regulations.

### Measures

#### Symptoms of depression

The 9-item Patient Health Questionnaire was used to assess patients’ symptoms of depression. Scores range from 0 to 27, with higher scores indicating more severe depressive symptoms. The PHQ-9 has been shown to have acceptable validity and reliability in individuals with heart failure^13^. In this study, a Cronbach’s alpha coefficient of 0.86 was found.

#### Symptoms of anxiety

Symptoms of anxiety were evaluated using the 7-item Generalized Anxiety Disorder (GAD-7), with potential scores ranging from 0 to 21, with higher scores indicating more severe symptoms of anxiety. The instrument has been shown to be a valid and reliable measure of anxiety^14^. In this study, a Cronbach’s alpha coefficient of 0.90 was found.

#### Quality of life

Quality of life was measured using the 12-item Kansas City Cardiomyopathy Questionnaire QoL subscale. The 12-item KCCQ provides an overall summary score as well as scores for the subscales Physical Limitation, Symptom Frequency, QoL, and Social Limitation^15^. Given the potential overlap between the Physical and Social Limitation subscales of the KCCQ-12 and the Return to Function subscale of the FPAS, we used only the scores from the QoL subscale and considered them as a measure of participants’ global satisfaction with their lives. In this study, a Cronbach’s alpha coefficient of 0.71 for the QoL subscale score was found.

#### Device acceptance

The Florida Patient Acceptance Survey (FPAS) was used to evaluate participants’ acceptance of the LVAD. The FPAS is a self-report questionnaire that was originally developed to measure patients’ acceptance of implantable cardiac devices (i.e., pacemakers, implantable cardioverter defibrillators). It consists of 18 items scored on a 5-point Likert scale. A total score and separate scores for four subscales (Return to Function, Positive Appraisal, Device-Related Distress, and Body Image Concerns) can be determined^16^. The Return to Function subscale assesses the extent to which a patient has returned to adequately engage in occupational, social, and physical activities. The Device-related Distress subscale evaluates the psychological distress associated with the device, and the Positive Appraisal subscale refers to the benefits of having the device implanted. Finally, the Body Image Concern subscale refers to perceived body disfigurement and feelings of being attractive. Validity and internal consistency for both total scores and all subscales, have been established in individuals with heart failure and implantable cardiac devices, and the FPAS has been shown to have robust performance independently of the time since implant^16–18^. In this study, Cronbach’s alphas were found to be 0.78 for total scores and 0.58, 0.76, 0.74, and 0.72 for Return to Function, Positive Appraisal, Device-related Distress, and Body Image Concerns respectively.

#### Socio-demographic and clinical variables

Socio-demographic data were obtained from a structured questionnaire. Clinical data were obtained from medical records.

### Statistical analysis

We performed mean and modal imputations to substitute missing values (<3%) in the continuous and categorical variables respectively. The bivariate associations between patients’ acceptance of the LVAD (total scores and subscale scores), age, time since LVAD implant, and symptoms of depression, anxiety, and QoL were assessed using Pearson’s correlation. The statistical significance of the difference between the FPAS values recorded in our cohort and those reported in the literature was assessed using unpaired t-tests.

Linear regression models were used to investigate further the cross-sectional association between patients’ LVAD acceptance dimensions and the symptoms of depression, anxiety, and QoL. Patients’ age and time since LVAD implant were *a priori* selected as potential confounding variables based on prior literature^8,11,18,19^, and were added to regression models. To account for the possible confounding effect of the patients’ physical ability, dummy variables for NYHA functional classes I and III were derived (with NYHA class II as the reference category) and entered as predictor variables. Unpaired t-tests were computed with Graph-Pad prism vs 7.01. All other analyses were done using SPSS version 20. A p < 0.05 was considered significant. All data can be made available upon request.

## Results

Table 1 presents the characteristics of the 101 consecutive patients recruited for the study. The sample was primarily Caucasian (73.3%) and male (78.2%), with a mean age of 57.65. Most of the participants were in NYHA functional class II (61.4%), and the average time from the diagnosis of heart failure was 9.78 years. Sixty-five % of the patients originally received the device as a Destination Therapy (DT).Older patients were more likely to be implanted as DT. In fact while only half of the patients in each of the two lowest age tertiles (age 22–54 and 55–65) were implanted as DT, almost 100% of the patients in the highest age tertile (65 to 84) were implanted without the future perspective of heart transplant. The average time since LVAD implant was 19.48 months (SD = 15.37).

**Table 1:** Socio-demographic and Clinical Characteristics of the Sample (N = 101)

Variables	Mean (SD) or N (%)	Range
Age	57.65 (14.18)	22–84
Sex		
Male	79 (78.2)	
Ethnicity		
Caucasian	74 (73.3)	
Black/African American	22 (21.8)	
Asian/pacific islander	2 (2.0)	
Another race/multiracial	3 (3.0)	
Marital Status		
Married or living with a partner	75 (74.2)	
Separated or divorced	14 (13.9)	
Widowed	2 (2.0)	
Single/never married	10 (9.9)	
Education		
Less than high school	7 (6.9)	
High school graduate	31 (30.7)	
Some college	37 (36.6)	
College graduate	19 (18.9)	
Any post-graduate	7 (7.0)	
Occupational status		
Employed	8 (8.0)	
Unemployed	7 (6.9)	
Retired	45 (44.6)	
On disability	40 (39.6)	
Student	1 (1.0)	
Perceived income adequacy		
Totally inadequate	10 (9.9)	
Not very well	29 (28.7)	
Adequate	52 (51.5)	
Very well	10 (9.9)	
HF etiology		
Ischemic	45 (55.4)	
NYHA functional class		
I	15 (14.9)	
II	62 (61.4)	
III	23 (22.8)	
IV	1 (1.0)	
Years since HF diagnosis	9.78 (8.12)	.30–35
LVAD Strategy		
Destination therapy	66 (65.3)	
Bridge to transplantation	35 (34.7)	
Type of LVAD		
HVAD	17 (16.8)	
HeartMate II	73 (72.3)	
HeartMate III	11 (10.9)	
Months since LVAD Implant	19.48 (15.37)	2–69

HF, heart failure; LVAD, Left Ventricular Assist Device; NYHA, New York Heart Association.

Analysis of the psychosocial measures (Table 2) showed that the average total scores of both the PHQ-9 and the GAD-7 were below the cut-off point of 10 for clinically significant symptoms of depression and anxiety^20,21^, although a sizeable percentage of participants reported clinically significant depression (23.8%) and anxiety (12.9%). Ten % of the patients reported having self-harm ideation at least several days during the prior two weeks. No patient reported active suicidal ideation. The average score on the KCCQ QoL subscale was 56.38.

**Table 2:** Descriptive Statistics of Psychosocial Measures (N = 101)*

Variables	Mean (SD) or %	Range
PHQ-9^†^	6.12 (5.62)	0–20
PHQ-9 ≥ 10	23.8%	
Self-harm Ideation^‡^	9.9%	
GAD-7^†^	3.67 (4.69)	0–18
GAD-7 ≥ 10	12.9%	
KCCQ QoL^§^	56.38 (26.28)	0–100
Florida Patient Acceptance Survey^ǁ^		
Return to function	41.31 (21.88)	0–100
Device-related distress	37.93 (23.15)	0–100
Positive appraisal	78.53 (23.66)	0–100
Body image concerns	39.33 (29.92)	0–100
Total score	60.74 (16.18)	0–100

GAD-7, 7-items Generalized Anxiety Disorder; KCCQ, Kansas City Cardiomyopathy Questionnaire; PHQ-9, 9-items Patient Health Questionnaire.

*For all the psycho-social measures, the scale (and subscales) ranges are reported.

^†^ PHQ-9 and GAD-7: higher scores mean higher psychological distress.

^‡^Percentage of patients reporting self-harm ideation at least several days during the last two weeks (item 9 of the Phq-9).

^§^KCCQ QoL: higher scores mean better health-related status.

^ǁ^Florida Patient Acceptance Survey: for total score and for Return to function and Positive appraisal, higher scores mean better functioning. For Device-related distress and Body image concerns higher scores mean worse functioning.

With regard to LVAD acceptance dimensions, patients’ average device acceptance was 60.74. Analysis of the four subscales of the FPAS showed that the acceptance of the implanted device was driven by a significant positive appraisal of the LVAD (mean “Positive Appraisal” subscale score 78.53) and that this balanced body image concerns (“body image concerns” subscale score mean 39.33), and the perception that the LVAD restored appropriate functional levels only in part (“return to function” subscale mean score 41.31). The average reported device-related distress was 37.93.

Analysis of bivariate associations (Table 3) showed that overall LVAD acceptance and almost all its subscales were associated with both the PHQ-9 and the GAD-7 total scores, as well as with the KCCQ QoL subscale scores. Specifically, a higher acceptance of the LVAD (FPAS total score) was associated with lower symptoms of depression (r = −0.50, p < 0.001) and anxiety (r = −0.50, p < 0.001) and higher perceived QoL (r = 0.47, p < 0.001). However, among the FPAS subscales, only the “Return to Function”, “Device-Related Distress” and “Body image concerns” subscales were associated with lower symptoms of depression and anxiety, while the “Positive Appraisal” subscale scores were not associated with mood or QoL. The results also showed a high correlation between anxiety and depression (r = 0.81, p < 0.001). Regarding the covariates, increasing age was associated with fewer symptoms of depression (r = −0.20, p < 0.05) and with higher perceived QoL (r = 0.54, p < 0.001). A trend was also found for the association between increasing age and lower levels of anxiety (r = −0.19, p = 0.06). Increasing age was also associated with higher overall acceptance of the LVAD as measured by the FPAS total scores (r = 0.36, p < 0.001), and with lower body image concerns as measured by the “Body Image Concerns” subscale of the FPAS (r = −0.22, p < 0.05). No significant association was found between months since LVAD implant and any of the measured patient-reported outcomes.

**Table 3:** Correlation between Covariates, Symptoms of Depression and Anxiety, Quality of Life, and Acceptance of the LVAD

Measures	1	2	3	4	5	6	7	8	9
1. Age	—								
2. Months since LVAD implant	0.16	—							
3. PHQ-9	−0.20*	0.03	—						
4. GAD-7	−0.19	0.12	0.81***	—					
5. KCCQ QoL	0.54***	−0.03	−0.57***	−0.47***	—				
6. Return to function	0.27**	−0.03	−0.47***	−0.42***	0.52***	—			
7. Device-related distress	−0.32**	0.07	0.48***	0.55***	−0.45***	−0.49***	—		
8. Positive appraisal	0.14	−0.07	−0.09	−0.06	0.01	0.18	−0.03	—	
9. Body image concerns	−0.22*	0.06	0.28**	0.27**	−0.26**	−0.39***	0.45***	−0.11	—
10. FPAS total scores	.36***	−0.08	−0.50***	−0.50***	0.47***	0.76***	−0.78***	0.50***	−0.65***

FPAS, Florida Patient Acceptance Survey; GAD-7, 7-item Generalized Anxiety Disorder; KCCQQoL, Kansas City Cardiomyopathy Questionnaire QoL; LVAD, Left Ventricular Assist Device; PHQ-9, 9-item Patient Health Questionnaire.

*p < 0.05 **p < 0.01 ***p < 0.001.

To further investigate the relationship between device acceptance and depression, anxiety, and QoL we built linear regression models and studied the relationship of the different components of the FPAS with the 3 variables (Table 4). This analysis confirmed the presence of a strong relationship between FPAS scores and depression (R^2^ = 0.30, F (4, 96) = 10.27, p < 0.001), anxiety (R^2^ = 0.33, F(4, 96) = 12.02, p < 0.001) and QoL (R^2^ = 0.33, F(4, 96) = 11.62, p < 0.001) respectively. The relationship between the FPAS, anxiety, depression, and QoL was mainly driven by a significant influence of the dimensions of “Return to function” and “Device-related distress”. The dimension “Return to function” had the largest impact on QoL (β = 0.41, p < 0.001) and had a negative relationship with depression (β = −0.30, p < 0.01) and anxiety (β = −0.20, p < 0.05). The dimension “Device-related distress” had the largest impact on anxiety (β = 0.46, p < 0.001) but was also associated with depression (β = 0.32, p < 0.01) and QoL (β = −0.26, p < 0.05). The regression analysis did not confirm the relationship between the “Body image concerns” subscale score and depression, anxiety or QoL (β between 0.01 and 0.08, not statistically different from 0).

**Table 4:** Unadjusted Linear Regression Analyses of Symptoms of Depression, Symptoms of Anxiety, and Quality of Life on Device Acceptance Dimensions

Predictors	Depression	Anxiety	QoL
	β	β	β
Return to function	−0.30**	−0.20*	0.41***
Device-related distress	0.32**	0.46***	−0.26*
Positive Appraisal	−0.02	−0.01	−0.08
Body Image Concerns	0.02	−0.02	0.01
Total R^2^	0.30***	0.33***	0.33***
Corrected Total R^2^	0.27***	0.31***	0.30***

R^2^, Variance; Corrected Total R^2^,Variance corrected for the number of predictors; β, Standardized regression coefficient. QoL, Quality of Life.

*p < 0.05 **p < 0.01 ***p < 0.001.

After accounting for the effects of age and time since LVAD implant, the only dimensions of the FPAS correlating with patients’ emotional distress and QoL were the “Return to function” and the “Device related distress” dimensions (Table 5). A lower score on “Return to function” was associated with a higher level of depression (β = −0.30, p < 0.01) and anxiety (β = −0.21, p < 0.05) and with lower QoL (β = 0.36, p < 0.001). The dimension “Device-related distress” had a strong influence on both depression (β = 0.32, p < 0.05) and anxiety (β = 0.45, p < 0.001). After adjusting for covariates, the relationship between “Device-related distress” and QoL was no longer statistically significant (β = −0.15, p = 0.11).

**Table 5:** Linear Regression Analyses Predicting Symptoms of Depression, Symptoms of Anxiety, and Quality of Life from Device Acceptance Dimensions, Adjusted for Age and Time since LVAD Implant

Predictors	Depression	Anxiety	QoL
	β	β	β
Return to function	−0.30**	−0.21*	0.36***
Device-related distress	0.32**	0.45***	−0.15
Positive appraisal	−0.02	0.01	−0.13
Body image concerns	0.01	−0.02	0.03
Total R^2^	0.30***	0.34***	0.49***
Corrected Total R^2^	0.26***	0.30***	0.45***

R^2^,variance; Corrected Total R^2^,Variance corrected for the number of predictors; β, Standardized regression coefficient; QoL, Quality of Life; LVAD, Left Ventricular Assist Device.

*p < 0.05 **p < 0.01 ***p < 0.001.

As a further sensitivity analysis, we tested the effect of adding the NYHA functional class to the linear regression models and confirmed that correcting for NYHA class did not significantly change our findings (Supplementary Table 1).

## Discussion

This is by far the largest study assessing body image concerns and device acceptance in LVAD recipients, and one of the largest studies assessing psychological distress in a population of real-world continuous flow LVAD recipients. Age and gender distributions in our patient population were roughly comparable to those reported in the most recent Interagency Registry for Mechanically Assisted Circulatory Support (INTERMACS) and European Registry for Patients with Mechanical Circulatory Support (EUROMACS) reports^22,23^. The study confirmed some findings from prior smaller studies^3–5^ and produced some novel and, in some cases, unexpected findings.

### Confirmation and Extension of Prior Work

First, we found that in the outpatient setting most patients living with an LVAD had a perceived QoL comparable to that observed in heart failure patients living without an LVAD (56.38 ± 26.28 vs 54.4 ± 24.1)^24^. This finding is consistent with data collected in clinical trials on LVAD^25^ and it supports the notions that: 1) LVAD support in the outpatient setting is characterized by better patient-reported QoL than among hospitalized LVAD patients; 2) LVAD support is associated with lower QoL than heart transplant^26^.

Second, we found that increasing age correlates with higher perceived QoL. This is consistent with what was previously observed in a retrospective analysis of the INTERMACS registry^27^.

Third, patients in our cohort reported a positive evaluation of the device as measured by the FPAS positive appraisal subscale, although the mean positive appraisal score for the LVAD was slightly lower than that previously reported in ambulatory heart failure patients for ICDs (78.53 ± 23.66 in our study vs 90.3 ± 18.5 in ICD recipients^28^, p < 0.01, Table 2). At the same time, patients reported significant device-related distress and body image concerns, far greater than those reported from patients who received ICDs (“device related distress” subscale score 37.93 ± 23.15 in our study vs 15.6 ± 20.3 in ICD recipients^28^, p < 0.001; “body image concerns” subscale score 39.33 ± 29.92 in our study vs 10.6 ± 22.2 in ICD recipients^28^, p < 0.001). Such findings are consistent with those reported in a smaller study in which LVAD recipients reported a positive global perception of their LVAD in spite of several negative somatic and QoL consequences^11^. In this regard, it should be noted that most of the participants in the present study were married or living with a partner. This may at least partially explain their ability to develop a positive evaluation of the device in spite of relevant difficulties, given the widely recognized supportive role played by partners in dealing with stressful conditions such as chronic illness^29^.

Fourth, we found clinically significant levels of depression and anxiety in 24% and 13% of patients respectively, with a small but non-negligible percentage of participants reporting at least occasional suicidal or self-harm ideation. These percentages are comparable to those reported in previous smaller studies of LVAD recipients and among individuals treated medically for heart failure^6,7,30^. It bears emphasis that while the existing literature on very small cohorts suggests a significant decrease over time in symptoms of depression and anxiety^2,3,6^, we did not find a significant association between time since LVAD implant and measures of psychosocial distress (Table 3). Additionally, percentages of patients with clinically relevant symptoms of psychological distress found in the current study are higher than those reported by research focusing on symptoms severity of anxiety and depression following a heart transplantation, where less than 8% and 6% of patients suffered from moderate to strong symptoms of depression and anxiety respectively^31,32^.

### Novel and Unexpected Findings

Our bivariate analysis showed a strong correlation between device acceptance and both psychological distress and QoL (Table 3) thus supporting our hypothesis. This is a novel finding in patients implanted with an LVAD but it is in line with the findings of previous studies that analyzed acceptance of implantable cardioverter defibrillators (ICDs)^18^ and is consistent with related, small scale studies (<34 patients) showing that in LVAD recipients distress is characterized by feelings of being useless and losing control over one’s life^6^.

In addition, our regression analyses highlighted the “Return to function” and “Device-related distress” domains of “device acceptance” as the major drivers of the correlations between device acceptance and depression/anxiety (Table 4). Importantly, the correlation remained significant even after adjusting for time since LVAD implant and patients’ age (Table 5).

Furthermore, the patients in our cohort reported strong concerns about their body image (as assessed by the “Body Image Concerns” subscale of the FPAS). The recorded average score in the Body Image Concern subscale of the FPAS was in fact 39.33 ± 29.92, a value markedly higher than that of ambulatory heart failure patients who received ICDs (10.6 ± 22.2, p < 0.01)^28^. Interestingly, contrary to our expectations, the regression analyses showed no correlation between the “body image concerns” dimension of “device acceptance” and psychological distress or QoL, when simultaneously considering the other device acceptance dimensions and relevant covariates (Tables 4 and 5). There is basically no prior data concerning body image in LVAD patients, with only two published studies that included a total of 15 patients. Chapman *et al*. performed a retrospective qualitative study on 6 LVAD recipients after transplantation or explanation and noted the feeling of being physically and psychologically scarred was a key theme characterizing the experience of LVAD recipients^12^. Marcucilli *et al*. studied 9 LVAD recipients and concluded that patients are able to develop long term psychological adaptive responses to a number of LVAD-related consequences as they recognize the life-saving role of the device^10^. Our findings are consistent with this latter notion.

Finally, the fact that recipients’ age correlated with “perceived return to function” and “device-related distress” (Table 3) is also an important novel finding. It is consistent with our finding of a negative relation between age and depression as measured by the PHQ-9 and a positive relation with perceived QoL as measured by KCCQ-12 QoL subscale. It is also consistent with psychological distress data recorded in stable heart failure patients living without an LVAD and with QoL data recorded in patients implanted with an LVAD^19,27^. This observation suggests that younger patients are more likely to experience emotional distress and poor QoL in the context of prolonged circulatory support, while elderly patients might be more likely to live happily on prolonged LVAD support. This stands to reason, as elderly patients supported with an LVAD might be more likely to achieve a functional capacity similar to that of their peers while most young patients, despite successful mechanical circulatory support, are likely to remain with significant functional limitations when compared with the general population in their age range.

### Limitations

The present study has several limitations. First, it is a cross-sectional study and the direction of causality between the variables cannot be determined. Second, we assessed patient acceptance using the Florida Patient Acceptance Survey. While this scale has been validated in patients living with heart failure, it was developed for the study of patients receiving small implantable cardiac devices and this is the first time it is used to assess device acceptance in LVAD recipients. Third, this is a single center study and therefore most of the participants included in this study come from the same region and received LVAD-related care from the same medical team. Fourth, we did not collect data on comorbidities. Fifth, in our study cohort, younger patients were less likely to receive LVAD as destination therapy than their older counterparts.This finding is in line with prior work^22^ showing that bridge to transplantation is the most used LVAD strategy among younger patients (aged less than 65) but it might have caused at least a portion of the increase in patient reported psychological distress noticed in younger subject. In fact, the existing literature supports the notion that living in the continuous expectation of a major life changing event such as organ transplant can be *per se* a trigger of psychological distress^33–35^.

## Conclusions

This study shows that (1) about a quarter of real-world stable patients living with an LVAD experience clinically relevant levels of depression or anxiety and that (2) poor device acceptance is associated with higher levels of psychological distress. Moreover, we found that: (3) perceived return to function is a major determinant of device acceptance; (4) body image concerns are not a major driver of device acceptance; (5) younger age correlates with poor device acceptance and high levels of psychological distress.

Since symptoms of psychological distress have been shown to have a negative impact on self-care performance and clinical outcomes in patients with heart failure and cardiac disease^36–38^, our findings point at device acceptance and perceived return to function as potential therapeutic targets to improve clinical outcomes in LVAD recipients. Moreover, our work highlights younger LVAD recipients as a population at especially high risk for poor patient-reported outcomes and challenges the routine implant LVADs in young patients who are not candidates for heart transplantation.

Psychosocial interventions based on a cognitive-behavioral approach and focusing on patients’ learning of specific coping skills have showed to be useful in managing the distress related to chronic illness^39^. Further studies will be needed to test the ability of psychological interventions to promote better adjustment to LVAD implant and improve the efficacy of this rapidly expanding therapy.

## Supplementary information

Supplementary material
