# Supplementary material for: Relationship between device acceptance and patient-reported outcomes in Left Ventricular Assist Device (LVAD) recipients
Source: Sci Rep. 2019 Jul 25;9:10778. doi: 10.1038/s41598-019-47324-z (PMC6658659; doi:10.1038/s41598-019-47324-z)
Supplement: Supplementary file 1 — Supplementary material [file 41598_2019_47324_MOESM1_ESM.docx]

**Relationship between device acceptance and patient-reported outcomes in Left Ventricular Assist Device (LVAD) recipients**

Crispino Tosto PhD^+1^, Luigi Adamo MD PhD*^+2^, Heidi Craddock NP^2^, Maria Di Blasi PsyD^1^, Rosario Girgenti PsyD^3^, Francesco Clemenza MD^3^, Robert M. Carney PhD^4^, Gregory Ewald MD^2^

^1^From the University of Palermo, Department of Psychological and Educational Sciences, Palermo, Italy

^2^From the Cardiovascular Division, Department of Medicine, Washington University School of Medicine St. Louis, MO, USA

^3^From the Heart Failure Unit, ISMETT, Palermo, Italy

^4^From the Department of Psychiatry, Washington University School of Medicine, St Louis, MO, USA

^+^these authors contributed equally to this work

| **Supplementary Table 1. Linear Regression Analyses Predicting Symptoms of Depression, Symptoms of Anxiety, and Quality of Life from Device Acceptance Dimensions, Adjusted for Age, Time since LVAD Implant, and NYHA Functional Class** | | | | | | | | |
| --- | --- | --- | --- | --- | --- | --- | --- | --- |
|  | Depression | |  | Anxiety | |  | QoL | |
| Predictors | β | |  | β | |  | β | |
| Return to function |  | ­-0.27* |  |  | ­-0.19^b^ |  |  | ­0.36*** |
| Device-related distress |  | ­0.34** |  |  | ­0.46*** |  |  | ­-0.15 |
| Positive appraisal |  | ­-0.02 |  |  | 0.01 |  |  | ­-0.13 |
| Body image concerns |  | ­0.02 |  |  | ­-0.02 |  |  | ­0.03 |
| Total R^2^ | 0.31*** |  |  | 0.34*** |  |  | 0.49*** |  |
| Corrected Total R^2^ | 0.26*** |  |  | 0.29*** |  |  | 0.44*** |  |
| NYHA, New York Heart Association; R^2^, Variance; Corrected Total R^2^, Variance corrected for the number of predictors; β, Standardized regression coefficient; QoL, Quality of Life; LVAD, Left Ventricular Assist Device; NYHA, New York Heart Association.  * p< 0.05 ** p<0.01 *** p < .001 ^b^ p=0.07 | | | | | | | | |
